# Energy-Dispersive X-ray Absorption Spectroscopy with an Inverse Compton Source

**DOI:** 10.1038/s41598-020-65225-4

**Published:** 2020-05-29

**Authors:** Juanjuan Huang, Benedikt Günther, Klaus Achterhold, Yi-tao Cui, Bernhard Gleich, Martin Dierolf, Franz Pfeiffer

**Affiliations:** 1 Department of Physics, Technical University of Munich, James-Franck-Straße 1, 85748 Garching, Germany; 2 Munich School of BioEngineering, Technical University of Munich, Boltzmannstraße 11, 85748 Garching, Germany; 3 Japan Synchrotron Radiation Research Institute, 1-1-1 Kouto, Sayo-gun, Hyogo, 679-5198 Japan; 4 Department of Diagnostic and Interventional Radiology, Klinikum Rechts der Isar, Technical University of Munich, 81675 Munich, Germany

**Keywords:** Characterization and analytical techniques, X-rays, Condensed-matter physics, Techniques and instrumentation

## Abstract

Novel compact x-ray sources based on inverse Compton scattering can generate brilliant hard x-rays in a laboratory setting. Their collimated intense beams with tunable well-defined x-ray energies make them well suited for x-ray spectroscopy techniques, which are typically carried out at large facilities. Here, we demonstrate a first x-ray absorption spectroscopy proof-of-principle experiment using an inverse Compton x-ray source with a flux of >10^10^ photons/s in <5% bandwidth. We measured x-ray absorption near edge structure and extended x-ray absorption fine structure at the silver K-edge (~25.5 keV) for a series of silver samples. We propose an energy-dispersive geometry specifically adapted to the x-ray beam properties of inverse Compton x-ray sources together with a fast concentration correction method that corrects sample inhomogeneities very effectively. The combination of our setup with the inverse Compton source generates x-ray absorption spectra with high energy resolution in exposure times down to one minute. Our results unravel the great benefit of inverse Compton scattering sources for x-ray absorption techniques in a laboratory environment, especially in the hard x-ray regime, which allows to probe absorption edges of high Z materials.

## Introduction

X-ray absorption spectroscopy (XAS) is an element-selective spectroscopic method which can probe the chemical surroundings around an atom of interest. More specifically, this technique can provide fingerprint information about the oxidation state, site symmetry, spin state, and thus is widely used in various research fields. Compared to other common x-ray techniques such as x-ray crystallography, XAS is not limited to crystalline or otherwise ordered samples, but is also applicable to disordered systems in different phases^1^. Among XAS, x-ray absorption near-edge structure (XANES) is sensitive to transitions from bound electronic core orbitals to unoccupied electronic orbitals which reveals the electronic structure. In contrast, extended x-ray absorption fine structure (EXAFS) is sensitive to local geometric structures on an atomic scale due to photoelectron interference occuring in this region^2,3^.

With increasing availability of synchrotron radiation from the 1970s, XAS has become widely applicable to different research fields^4^. However, the limited access to and high cost of large-scale synchrotron facilities inhibit the wide-spread use of XAS as part of standard laboratory workflows on a daily basis. Although these days the performance of XAS implemented with x-ray tubes has greatly improved^5–7^, the low brilliance when using bremsstrahlung results in long acquisition times, restricting its applications to only a few certain samples and research subjects.

This limitation can be overcome by recent developments in novel compact x-ray sources based on laser-produced plasma (LPP)^8^, high-harmonic generation (HHG)^9,10^, betatron radiation^11^ or inverse Compton scattering (also called Thomson scattering)^12–14^. Among them, static or ultrafast XAS has recently been demonstrated with HHG^15,16^ and betatron radiation^17^ with x-ray energies lower than 2 keV, and LPP^18^ with x-ray energies lower than 10 keV.

Even at large facilities, some synchrotrons cannot measure high-energy XAS due to limited storage ring sizes, which results in limited electron energies and x-ray energies. For example, 5 out of 12 facilities listed by the Wayforlight initiative of the European synchrotrons^19^ cannot measure XAS with x-ray energies >10 keV. In this hard x-ray regime, inverse Compton scattering (ICS) sources are very advantageous, which can provide intense, pulsed x-rays with a narrow, tunable spectrum. The strong field of an intense laser is utilized instead of a classical permanent magnet undulator to wiggle the electrons^12^. Thereby decreasing the undulator period by four orders of magnitude, electron energies on the order of tens of MeV are sufficient to generate high-energy hard x-rays. This enables very compact footprints of inverse Compton sources, e.g. 7 × 3 m for the one installed at the Munich Compact Light Source (MuCLS)^20^. Consequently, many projects are currently ongoing worldwide to develop ICS sources^21–26^. Apart from some proof-of-principle work in protein crystallography^27^, ICS sources have been used mainly in x-ray imaging related research^28–32^ so far.

Here, we demonstrate the first XAS study with an ICS source. We present energy-dispersive XAS at the MuCLS utilizing a relatively simple setup; a slightly bent silicon wafer together with an x-ray camera. The energy resolution and energy range for XAS measurements can be tuned by adjusting the crystal curvature, asymmetry angle or choosing different diffraction orders. The setup allows us to obtain XAS spectra with a high energy resolution at exposure times down to one minute. We carried out XANES and EXAFS measurements at the silver K-edge with a series of different silver compounds, which yield spectra with a quality comparable to synchrotron data. Therefore, we believe that for this hard x-ray regime >10 keV, our approach is among the most efficient XAS acquisition schemes implemented in a laboratory setting. In conjunction with the proposed experimental approach, we also introduce a feasible, fast concentration correction method without using a focused x-ray beam, which corrects spatial sample inhomogeneities very effectively. This facilitates the measurement of inhomogeneous samples such as a typical XAS pellet, which is very difficult to handle both at synchrotrons and in laboratories using energy-dispersive geometries^6,33^.

## Experimental setup

### The Munich Compact Light Source

The MuCLS is a laboratory x-ray facility^20^ with two end-stations equipped with multi-purpose x-ray experimental setups^30^. The ICS source of the MuCLS is manufactured by Lyncean Technologies Inc., Fremont, USA and this commercial ICS source is available at a cost of a few percent of a large synchrotron facility^34^. The investment for an entire facility similar to ours, including radiation shielding, infrastructure and a beamline with two endstations in addition to the source, is in the range of 12–18 million Euro, but depends strongly on the specific local conditions. A single endstation exclusively dedicated to XAS with the setup described in the manuscript could be constructed for ~100 kEUR, with ~50 kEUR for a small radiation shielding (lead) enclosure and ~50 kEUR for motorized stages, a motor controller, a detector and PCs. However, in this work, the only additional component for the proposed energy-dispersive XAS setup based on our already existed imaging application setups was the optics – a 200 μm thick Si<100> wafer, which costs less than 100 EUR.

**Figure 1:**
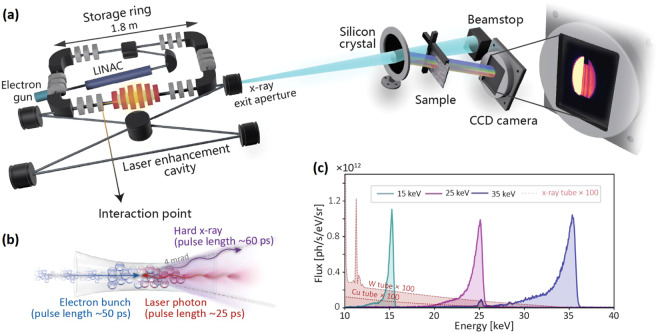
(**a**) Energy-dispersive x-ray absorption spectroscopy (XAS) setup at the Munich Compact Light Source (MuCLS). (**b**) Hard x-rays are produced in the process of inverse Compton scattering (ICS). (**c**) Three exemplary MuCLS source spectra at 15, 25, 35 keV configurations with bandwidths of 3.0% 3.6% and 4.3% (FWHM), respectively. The small peak visible for the 35 keV spectrum at 25 keV is due to the Sn-K*α* fluorescence line of the solder from the Amptek X-123 detector. For comparison, simulated x-ray tube spectra for W and Cu anodes are plotted (intensity scaled by a factor of 100).

The ICS source comprises a short linear electron accelerator (LINAC), a small electron storage ring^35^ and a laser system containing a high-finesse enhancement cavity (Fig. 1a). The electrons are generated in an RF-photocathode at a rate of 25 Hz and get accelerated to relativistic speed in the LINAC. Afterwards, the electrons are injected into a miniature storage ring, which has a circumference of 4.62 m. At the same time, two infrared (IR) laser pulses (Nd:YAG) are stored in a high-finesse bow-tie enhancement cavity (9.2 m length) with an average power of ~300 kW. At the interaction point (Fig. 1b), x-rays with a pulse length of ~60 ps are generated in a head-on collision of the electron bunch and the laser at a repetition rate of 64.9 MHz. Both, the electron bunch and the IR laser, are tightly focused at the interaction point, giving rise to x-ray source size of 50 μm rms. The divergence of the x-ray beam is confined to ~4 mrad by an aperture.

The x-ray energy on axis for a head-on collision of electron and laser pulse is given by Eq. (1). As the laser wavelength is fixed at 1064 nm, the x-ray energy can be tuned flexibly by adjusting the electron energy:

1 $${E}_{x}\approx 4{\left(\frac{{E}_{e}}{{m}_{e}{c}^{2}}\right)}^{2}{E}_{L},$$

where *E*_*x*_, *E*_*e*_ and *E*_*L*_ are the energies of x-ray, electron and laser, respectively, *m*_*e*_ is the electron rest mass, and *c* is the speed of light in vacuum. Currently, the x-ray energies available at the MuCLS range from 15 to 35 keV, corresponding to electron energies between 25 and 45 MeV. Figure 1c shows exemplary spectra at three different x-ray energy configurations, 15 keV, 25 keV and 35 keV (measured with an energy-dispersive detector, Amptek X-123, Amptek Inc., Bedford, USA). Also, a Monte Carlo simulation was done for x-ray tubes with Cu (30 kVp, 25 mA) and W anodes (35 kVp, 25 mA) using the PENELOPE^36^ package. In the simulation, a homogeneous anode-substrate is considered and the beam spot size is 10 μm. The anode-target angle and the take-off angle are 11 degrees and 25 degrees respectively. All other simulations parameters were left at the default values of the package. The full input parameter files used are provided as Supplementary information.

The x-ray properties such as x-ray flux and x-ray positions were measured by a specifically designed x-ray beam monitor^37^, which intercepts only a small fraction of the x-ray beam upstream. The intensity maximum of ~1 × 10^12^ photons/s/eV/sr is similar for all energy configurations, which is several orders of magnitudes higher than that of x-ray tubes at corresponding energies. The integrated flux and spectral bandwidth of the entire x-ray beam with ~4 mrad divergence quasi-linearly increase with x-ray energy, resulting in values of ~1 × 10^10^ photons/s, ~2 × 10^10^ photons/s and ~3 × 10^10^ photons/s with 3.0%, 3.6% and 4.3% bandwidth (full width at half maximum, FWHM), respectively. The bandwidth at each energy configuration is wide enough to cover the EXAFS energy range, but also narrow enough to prevent harmonic contamination which introduces high background in XAS spectra^33^.

### The energy-dispersive XAS setup

**Figure 2:**
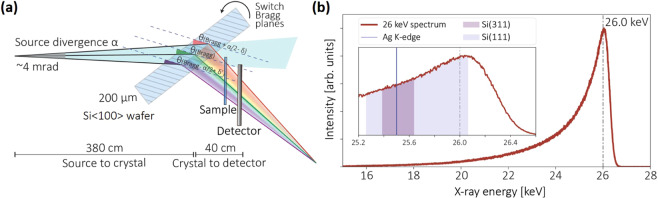
(**a**) Geometric illustration of the energy-dispersive setup. The ~4 mrad cone angle of the x-ray source together with a Laue crystal results in an energy gradient across the detector. (**b**) The source spectrum was tuned to 26 keV configuration  for Ag measurement (measured with an energy-dispersive detector, KETEK AXAS-D, KETEK GmbH, Munich, Germany). The inset shows a zoom-in of the spectrum. The colored regions indicate the energy ranges that can be covered by Si(311) and Si(111) diffracted beams, corresponding to XAS images in Fig. 3.

In our XAS experimental setup (see Fig. 2a), the main components besides the MuCLS are a slightly bent Si<100> crystal wafer (200 μm thick, Ø = 100 mm) in Laue geometry and a CCD camera (XIMEA xiRAY, XIMEA GmbH, Münster, Germany), with a fibre-optically coupled scintillator (Gd_2_O_2_S:Tb), a pixel size of 9 μm and a field-of-view (FOV) of 24 mm × 36 mm (horizontal × vertical). The Laue geometry (transmission geometry) rather than Bragg geometry (reflection geometry) was chosen as it prevents spectrum distortions at higher energies^38^. The measurements were carried out in ambient condition, as the absorption of high-energy x-rays by air is rather small. In contrast to most energy-dispersive XAS beamlines where samples are illuminated with a focused x-ray beam, we put the samples in close proximity behind the silicon crystal where they were illuminated with a relatively large x-ray beam. Therefore, the setup is realized without a sophisticated bender design, and can be very easily aligned. In addition, because of the unfocused geometry, not only the dispersive energy gradient is imaged, but also the 2d spatial information of the sample. In our XAS experiment, we measured compounds containing the element silver (Ag), whose K-edge is located at 25.5 keV. Therefore, we chose the MuCLS 26 keV-configuration (see Fig. 2b) with a FWHM of 1088 eV. The total flux of the entire x-ray beam with ~4 mrad divergence impinging on the crystal was ~1.2 × 10^10^ photons/s at the time of the experiment.

### Energy resolution & energy range tuning

The bending radius of the crystal can be tuned by slightly pushing its edges in the designed crystal holder. The beam size is correlated to the asymmetry angle – the angle between the Si wafer’s surface and the crystal plane. The energy range diffracted by the crystal is determined by the x-ray source divergence, bending curvature and crystal planes (see Eq. 5).

**Figure 3:**
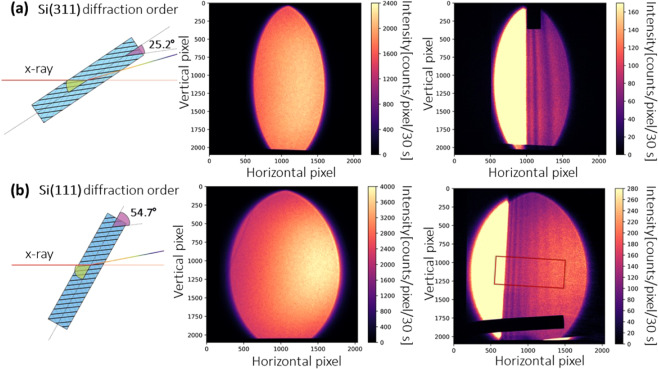
(**a**) The Si(311) and (**b**) Si(111) diffracted Laue beams with a 50 μm Ag foil in the beam. The diffraction order can be switched by simply rotating the Laue crystal. The total energy gradient range corresponds to the colored regions in the inset of Fig. 2b. The CCD camera can cover the whole Laue diffracted beam, which has an elliptical shape with an energy gradient encoded along the horizontal direction and spatial information at same energy encoded along the vertical direction. A strongly absorbing metal bar (Ø = 1.5 mm) was fixed together with the sample for alignment. The non-parallel absorption fringes are due to non-ideal bending of the crystal.

Figure 3 shows an example of the energy resolution and energy range tuning. Here, a bent Si<100> silicon wafer with bending radius of ~11 m was used. The transmitted intensity oscillations (visible as fringes) correspond to the absorption fine structure of Ag. Because of the smaller asymmetry angle, the Si(311) diffracted beam is narrower than the Si(111) diffracted beam. However, the energy range of the Si(311) diffracted beam is also much narrower, giving rise to an increase of energy resolution by a factor of about two. The maximum energy range and maximum x-ray beam size in horizontal direction for the Si(311) and Si(111) diffracted beams were ~250 eV, ~1000 pixels and ~780 eV, ~1500 pixels, respectively. The Si(311) diffraction order of this crystal was chosen for XANES measurement due to the higher energy resolution and appropriate energy range.

In this Laue geometry, different Bragg diffraction orders such as Si(311) and Si(111) diffraction orders can be quickly switched by simply rotating the crystal. Therefore, different energy resolutions and different energy ranges can be selected during a single experiment without changing the crystal, which results in a highly flexible XAS setup. This capability of tuning energy resolution and energy range without replacing the crystal is difficult to realize in other energy-dispersive beamlines.

## Results and Discussion

In this proof-of-principle experiment, we measured different Ag samples in different phases: 1) 5 M aqueous AgNO_3_ solution, 2) 50 μm thick Ag foil and 3) Ag_2_O (50 mg) powder pressed into a pellet with 100 mg BN.

### XANES data acquisition for homogeneous samples

**Figure 4:**
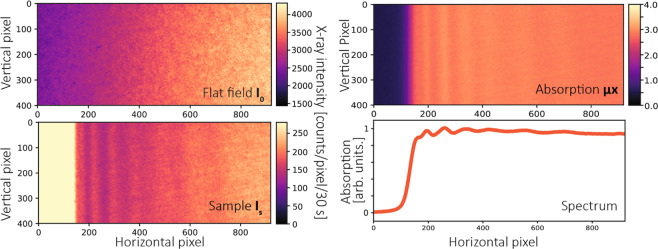
Data acquisition procedure for a Ag foil sample using Si(111) diffraction order. All images are cropped from the center region of the Laue diffracted beams using a rectangular region-of-interest (ROI) with a size of 920 × 400 pixels (horizontal × vertical), as marked in Fig. 3b. The x-ray image *I*_*s*_ shown here is the same data as shown in Fig. 3b. All the images are dark-current corrected and filtered by a 2d median filter.

For homogeneous samples such as the Ag foil and the AgNO_3_ solution, measurements were performed taking projections with the sample and without it (see Fig. 4). All images were dark-current corrected and filtered by a 2d median filter with a kernel size of 3 × 3 to remove “pepper and salt” like impulse noise^39^ without affecting spectral features. The measured 2d absorption images were calculated for each pixel according to Lambert-Beer’s law:

2 $$\mu x=-\,\mathrm{ln}\,\frac{{I}_{s}-{I}_{d}}{{I}_{0}-{I}_{d}}$$

where *μ* is the linear attenuation coefficient, *x* the thickness of the sample, *I*_*d*_ the dark current of the CCD detector, *I*_*s*_ and *I*_0_ the transmitted x-ray intensities with and without the sample.

In order to get the final spectrum, the image needs to be rotated so that all absorption fringes are vertical. If the absorption fringes are not parallel to each other, the image can be further warped using an affine transform (see Supplementary information). In this example (Fig. 4), a rectangular region-of-interest (ROI) with a size of 920 × 400 (horizontal × vertical) pixels was chosen. As a proof-of-principle example, we only present strictly necessary operations and thus keep the data as close to the original state as possible. Therefore, we did not apply the warping correction in Fig. 4, which may slightly degrades the final spectrum due to the nonparallel features.

A bigger ROI or polygonal ROI can also be chosen to even further reduce the acquisition time depending on demands of experiments. By averaging all the values along the vertical direction of the ROI, the spectrum can be obtained as a function of the horizontal pixel position. The pixel position is then calibrated to x-ray energy by second-degree polynomial fits to a reference spectrum, which was measured using quick XAFS (QXAFS) at SPring-8 (BL14B2, Si(111) double crystal monochromator). The same rotation angle, warping parameters and second-degree polynomial function can be further used for other unknown sample measurements containing elements with the same absorption edge.

### XANES data acquisition for inhomogeneous samples

**Figure 5:**
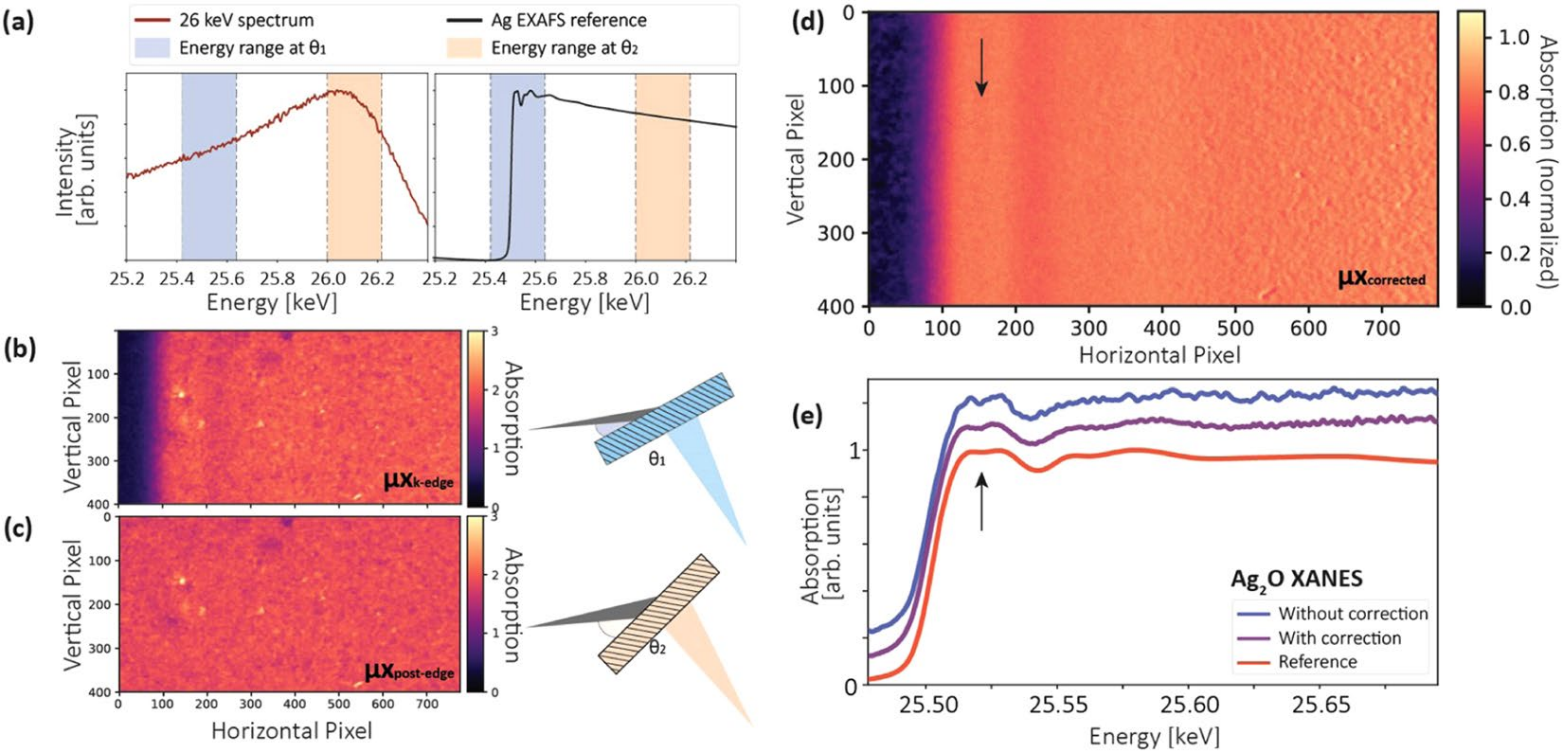
Concentration correction method for an inhomogeneous sample. A rectangular region-of-interest (ROI) with a size of 780 × 400 pixels was chosen for all images. (**a**) The source spectrum and a reference Ag_2_O extended x-ray absorption fine structure (EXAFS) spectrum. In addition to the absorption measured at the K-edge (blue energy range), another measurement was carried out at higher energies (orange energy range). (**b**) The cropped 2d absorption images for measurements with the Laue crystal at rotation angle *θ*_1_ and (**c**) rotation angle *θ*_2_. (**d**) The concentration-corrected image was calculated using the the local thickness extracted from the image recorded at *θ*_2_. (**e**) Compared with the Ag_2_O x-ray absorption near edge structure (XANES) spectrum without concentration correction (blue curve), the quality of the corrected data is greatly improved.

In the proposed geometry, the diffracted beam has a large footprint on the sample, e.g., ~8.7 mm × ~17.3 mm (horizontal × vertical) for the Si(311) diffracted beam in Fig. 3a. Therefore, sample inhomogeneity can pose a significant problem. This is because different energies are impinging on different locations of the sample. Fig. 5b shows a cropped 2d absorption image (780 × 400 pixels, horizontal × vertical) for the Ag_2_O powder pellet, for which most spectral features are non-distinguishable.

For energy-dispersive XAS implementations both at synchrotrons and with x-ray tubes, such a standard XAS pellet is usually quite difficult to handle. At laboratory setups^6,40^, x-rays are not focused, which gives rise to similar degradation as shown in Fig. 5b. At synchrotrons, though x-rays are focused, small-angle scattering from the pellet degrades a measured spectrum significantly (see example in ref. ^33^). Also, for this focusing geometry at a synchrotron, usually very accurate control of the crystal bending is needed and thus a more sophisticated bender design is required.

Here, we propose a fast concentration correction method without using a focused or monochromatic beam. This method was demonstrated with the Ag_2_O pellet (Ø = 13 mm): in addition to the absorption image (*μ*_*K*−*edge*_*x*) measured at the K-edge at a fixed Bragg angle (Fig. 5b), a second absorption image (*μ*_*post*−*edge*_*x*) was taken at a smaller Bragg angle (Fig. 5c) by rotating the Si wafer by 0.2 degrees (*θ*_2_ − *θ*_1_). This caused a shift of the energy range by ~600 eV to the post-edge region of the spectrum where no spectral features are present (Fig. 5a). Consequently, the attenuation coefficient in this region (*μ*_*post*−*edge*_) can be approximated by a linear function. Therefore, the corrected attenuation coefficient (shown in Fig. 5d) at each pixel, which is no longer affected by local thickness variations, can be extracted by:

3 $${\mu }_{corrected}=\frac{{\mu }_{K-edge}x}{{\mu }_{post-edge}x}$$

With the crystal rotation, there was a slight movement of the x-ray beam relative to the sample. Two images need to be aligned pixel-to-pixel. The mismatch of the alignment gives rise to the increased noise on the right of Fig. 5d. This can be further improved by better sample alignment or some further image post-processing such as non-linear registration or image filtering.

In principle, the second image at higher energies should be taken as far away in energy as possible from the K-edge absorption image to avoid spectral features in EXAFS. However, the energy range is limited by the 26 keV ICS source x-ray spectrum in this proof-and-principle experiment, and can be further increased by using a slightly higher source energy configuration.

Nevertheless, the resulting spectrum in this proof-and-principle experiment already shows great improvement compared to the uncorrected one (Fig. 5e), e.g., the tiny spectral feature (see arrows), which can not be unambiguously identified in the uncorrected spectrum, was well reconstructed during this process, demonstrating the effectiveness of the method.

### XANES results

**Figure 6:**
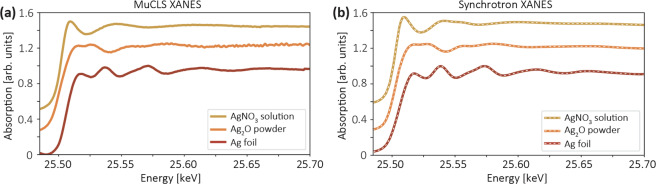
(**a**) Experimental XANES spectra of a series of Ag samples measured at the MuCLS. (**b**) Reference spectra taken at BL14B2, SPring-8.

Using the data acquisition scheme described before, we were able to measure different Ag samples in different phases and obtained the results shown in Fig. 6a in comparison with synchrotron spectra shown in Fig. 6b. The exposure time was 30 s for individual frames with and without samples and 10 frames were averaged for better statistics. However, all spectral features can be still well resolved using only one individual frame (see Supplementary information). All the spectra show excellent agreement with spectra measured at the synchrotron. The results shown here used the Si(311) diffraction order of a Si<100> wafer with a bending radius of ~11 m. The energy resolution was estimated to be ~3.8 eV, smaller than the lifetime broadening of the Ag K-edge (~6.5 eV)^41^. Therefore, all spectral features can be resolved well.

### EXAFS results

We also measured an EXAFS spectrum for the Ag foil. In order to increase the energy range, the presented EXAFS data was recorded using the Si(311) diffraction order of another Si<100> crystal with a different bending radius of ~21 m. This allowed to record a larger maximum energy range of ~460 eV in one image than with the crystal used for the XANES data (maximum energy range ~250 eV, bending radius of ~11 m). Again, we rotated the crystal by ~0.05 degree to extend the total energy range, corresponding to a shift of the maximum energy range of the whole Laue diffracted beam by ~150 eV. The total energy range we can cover is currently limited by the ICS source x-ray spectrum, which drops quickly after 26 keV. This can be improved in the future by using a slightly higher source energy configuration spectrum, e.g. with the peak intensity at 26.5 keV, and thereby using a larger rotation angle to cover a larger energy range in total.

**Figure 7:**
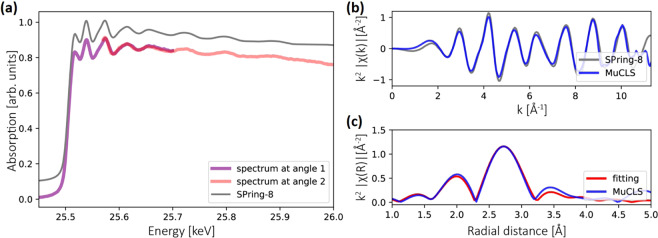
(**a**) The EXAFS of the Ag foil was measured by stitching two spectra measured with the Si(311) diffraction order. Both spectra have several distinct features in common. (**b**) The *k*^2^-scaled EXAFS function in k space. (**c**) Fourier transform of experimental EXAFS function and EXAFS fitting to the first shell.

The spectral overlap (for the cropped spectra, which are the purple and pink curves shown in Fig. 7a) is ~130 eV. We calibrated both spectra and combined them together. The limitation of the current method is that distinct features are needed in the second image and some spectrum overlap (e.g., at least one peak/feature in common) is required in order to stitch them together. Other data acquisition methods can also be used to measure EXAFS to overcome this limitation (see Supplementary information). The obtained spectrum and its EXAFS function in k-space are shown in Fig. 7a,b. The k^2^-weighted EXAFS function is Fourier transformed (see Fig. 7c) to get information about interatomic distances. By fitting to theory using the ARTEMIS software^42^, we extract a nearest-neighbor Ag-Ag distance of 2.87 Å. This value is smaller than the 2.89 Å determined by x-ray crystallography^43^, which can be explained by an asymmetric bond-length distribution in the first coordination shell of the Ag foil^44^.

## Conclusion

In summary, we demonstrate the first proof-of-principle XAS spectroscopy study with an inverse Compton scattering source. Their energy tunability, few percent bandwidth and for laboratory setups high brightness make ICS sources particularly suited for routine x-ray spectroscopy studies in laboratories.

Finally, apart from transmission mode XAS, ICS sources promise to be good candidates for fluorescence mode XAS, which is essential for diluted systems. Moreover, the pulse lengths of hard x-rays on the picosecond scale open a potential path for time-resolved studies investigating picosecond phenomena at high energies in a laboratory environment.

## Methods

### Crystal bending radius estimation

The bending radius of the crystal can be estimated from the experimentally measured energy range, e.g., the horizontal energy coverage of the elliptical Laue diffracted beam shown in Fig. 3.

For a perfectly flat crystal, the energy range given by a 4 mrad x-ray source divergence is:

4 $$\Delta {E}_{flat}={E}_{0}\cdot 4\,{\rm{mrad}}\cdot \,\cot ({\theta }_{Bragg})$$

where E_0_ is the x-ray central energy and *θ*_*Bragg*_ is the Bragg’s angle. The energy range for a slight crystal bending is:

5 $$\Delta {E}_{bent}={E}_{0}\cdot \mathrm{[4}\,{\rm{mrad}}-(\delta +\delta {\prime} )]\cdot \,\cot ({\theta }_{Bragg})$$

where *δ* and *δ*′ are the angular deviation caused by the local bending curvature (see Fig. 2a). The x-ray beam footprint *l* on the crystal is:

6 $$l=s/\sin (\alpha )$$

where *s* is the transverse x-ray beam size at the crystal-to-source distance, *α* the crystal rotation angle (*α* = *θ*_*Bragg*_ + *χ*, where *χ* is the asymmetry angle). The estimated radius of curvature *R* of the crystal is then:

7 $$R=l/(\delta +\delta {\prime} )$$

In the geometry used in this experiment, the slight curvature results in a smaller energy range and a wider Laue diffracted beam on the detector, thus increasing the energy resolution.

### Energy resolution estimation

**Figure 8:**
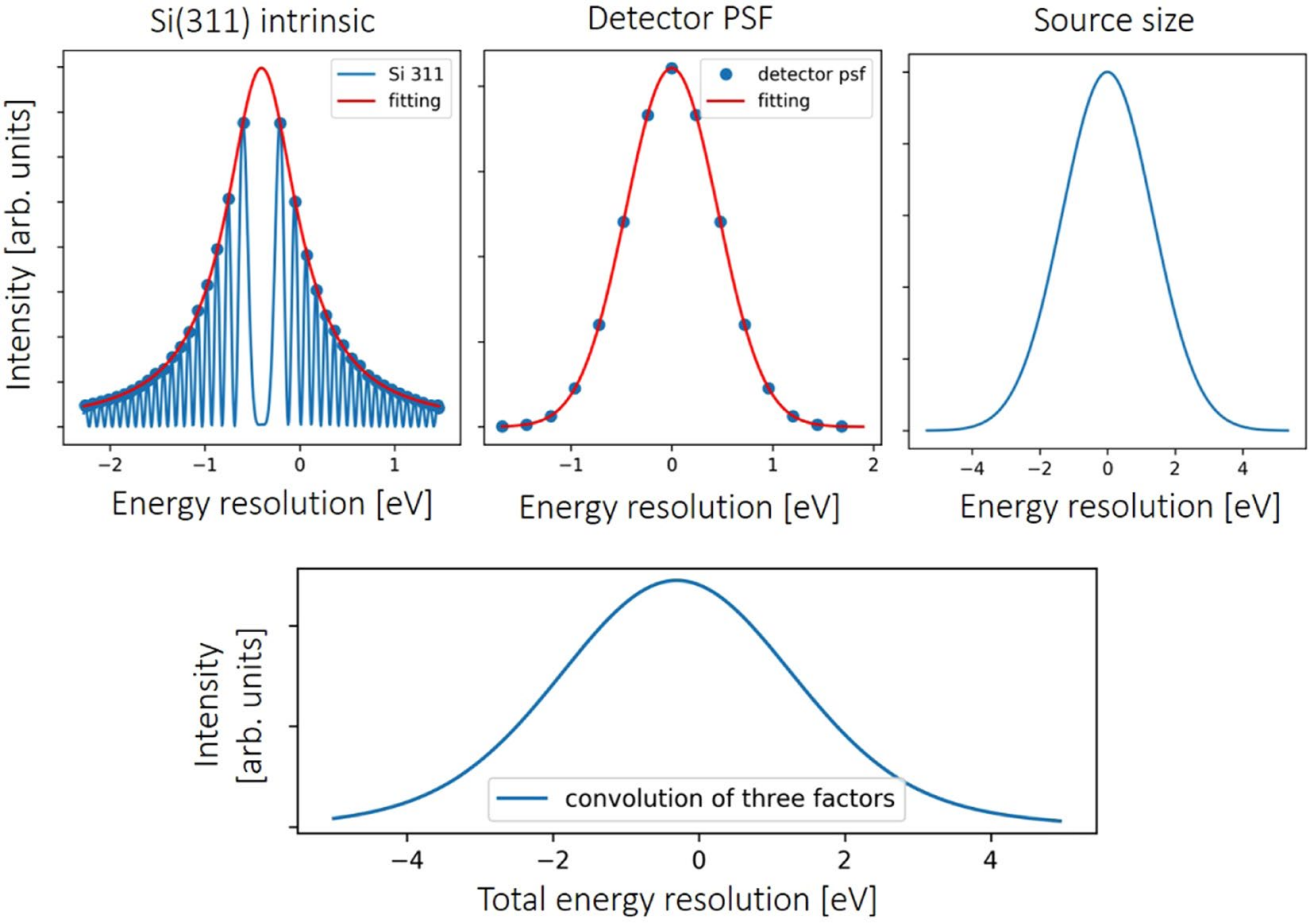
Energy resolution estimation for the Si(311) diffracted beam of a Si<100> crystal with a bending radius of ~11 m.

It is not very straightforward to determine energy resolution in energy-dispersive XAS. Therefore, the energy resolution is estimated by a convolution of the crystal’s intrinsic energy bandwidth, the detector point spread function (PSF) and the x-ray source size. Figure 8 shows the evaluation of the energy resolution for the crystal used to obtain the XAS spectra in the manuscript. The Laue crystal reflectivity was calculated using the XOP2.4 software^45^ and fitted by a Lorentzian function. The PSF function for the Ximea detector was determined by measuring a Siemens star test pattern. The x-ray source size was obtained from a knife edge projection image assuming a Gaussian source spot, which results in a sigma value of 50 μm. The final energy resolution was approximated by the FWHM value of the convolution profile of these three functions, giving an estimated energy resolution shown in Table 1. The spectral resolving power is defined as E/ΔE.

**Table 1:** The energy resolution and resolving power at 25.5 keV.

bending radius	diffraction order	intrinsic bandwidth	source size	detector PSF	energy resolution	resolving power
~11 m	Si(311)	0.9 eV	3.2 eV	1.1 eV	~3.8 eV	~6711
~11 m	Si(111)	3.5 eV	6.5 eV	2.2 eV	~9.0 eV	~2833
~21 m	Si(311)	0.9 eV	6.5 eV	2.2 eV	~7.1 eV	~3591

The presented values are the FWHM values for the corresponding functions.

### EXAFS fitting

The EXAFS fitting was performed using the ARTEMIS software^42^. The EXAFS equation is^46^:

$$\chi (k)=\sum _{i}\,\frac{{N}_{i}{S}_{0}^{2}}{k{R}_{i}^{2}}{F}_{i}(k){e}^{-2{R}_{i}/{\lambda }_{i}(k)}{e}^{-2{k}^{2}{\sigma }_{i}^{2}}\,{\sin }[2k{R}_{i}+{\Phi }_{i}(k)]$$

where k is the photoelectron momentum index, $k=\sqrt{2m(E-{E}_{0})/{\hslash }^{2}}$, *N*_*i*_ the coordination number, ${S}_{0}^{2}$ the amplitude reduction factor, *R*_*i*_ the interatomic distance, *F*_*i*_ the photoelectron scattering amplitude, *λ*_*i*_(*k*) the photoelectron inelastic mean free path, ${e}^{-2{k}^{2}{\sigma }_{i}^{2}}$ the effective Debye-Waller factor, and i notifies the respective atom in the modeling system. Both the *F*_*i*_ and Φ_*i*_(*k*) depend on the atomic number. The coordination number and possible scattering paths were based on the Ag crystallographic data^43^. Multiple-scattering paths were not considered and two direct paths for nearest neighbors were included in the fitting process. Therefore, four parameters are fitted, ${S}_{0}^{2}$, *R*_*i*_, ${\sigma }_{i}^{2}$, *E*_0_ and are summarized in Table 2. For unknown samples measured at the same condition, the same ${S}_{0}^{2}$ value should be fixed. Then the coordination number can be determined from the fitting instead.

**Table 2:** EXAFS fit results.

shell	CN	${{\bf{S}}}_{{\bf{0}}}^{{\bf{2}}}$	*R*_*i*_ [Å]	*σ*^2^ [Å^2^]	*E*_0_ [eV]	R [%]
Ag-Ag	12	0.73	2.87	0.0094	−0.51	1.27

The residual factor is calculated by:

$$R=\sqrt{\frac{\sum \,({k}^{2}{\chi }_{{\exp }}-{\chi }_{calc})}{{({k}^{2}{\chi }_{{\exp }})}^{2}}}$$

where *χ*_*exp*_ and *χ*_*calc*_ is the experimentally measured and theoretically fitted EXAFS oscillation, respectively.

## Supplementary information

Supplementary Information.

Suppplementary Information 2

Suppplementary Information 3
