# Supplementary material for: Energy-Dispersive X-ray Absorption Spectroscopy with an Inverse Compton Source
Source: Sci Rep. 2020 May 29;10:8772. doi: 10.1038/s41598-020-65225-4 (PMC7260230; doi:10.1038/s41598-020-65225-4)
Supplement: Supplementary file 1 — Supplementary Information. [file 41598_2020_65225_MOESM1_ESM.pdf]

Supplementary Information for

Energy Dispersive X-ray Absorption Spectroscopy with an Inverse Compton Source

Juanjuan Huang[a,b,*], Benedikt Günther[a,b], Klaus Achterhold[a,b], Yi-Tao Cui[c], Bernard Gleich[b], Martin Dierolf[a,b,*] and Franz Pfeiffer[a,c]

[a] Department of Physics, Technical University of Munich, James-Franck-Straße 1, 85748 Garching, Germany
[b] Munich School of BioEngineering, Technical University of Munich, Boltzmannstraße 11, 85748 Garching, Germany
[c] Japan Synchrotron Radiation Research Institute,1-1-1 Kouto, Sayo-gun, Hyogo 679-5198 Japan
[d] Department of Diagnostic and Interventional Radiology, Klinikum Rechts der Isar, Technical University of Munich, 81675 Munich, Germany

S1 Possible elements which can be investigated with the MuCLS

As stated in the main manuscript, the energy of our ICS source can be tuned freely in the range from 15 keV to 35 keV which can cover the absorption edges highlighted in the following figure:

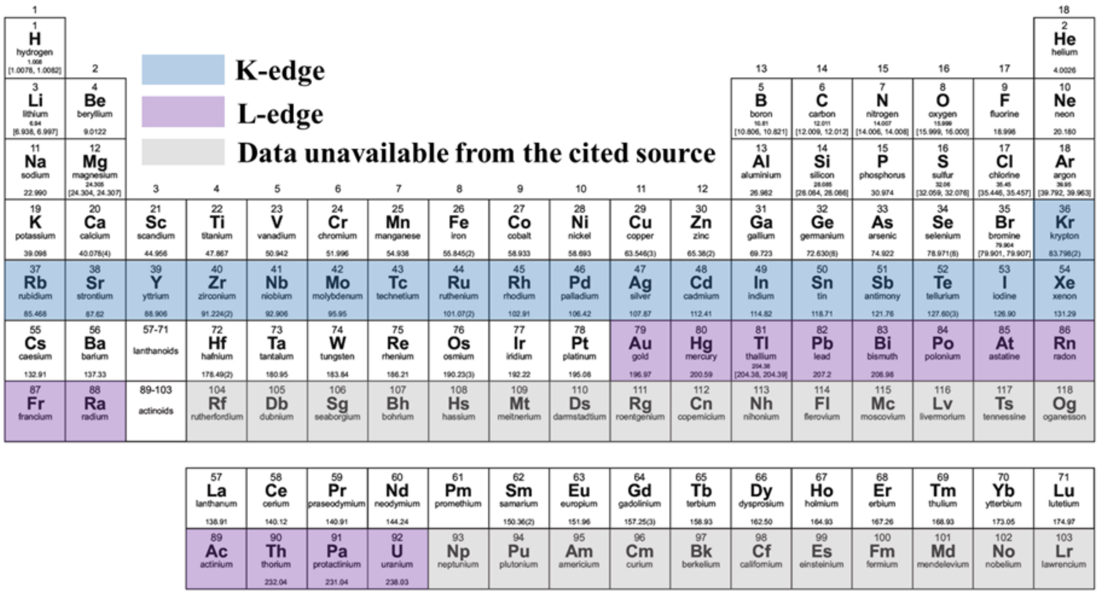

Figure S1 Absorption edges that can be covered by the energy range (15 keV - 35 keV) available at the MuCLS. The absorption edge data is from [1] and the periodic table is adapted from [2]

S2 Warping correction

If the crystal bending is not perfectly, one may observe that the absorption fringes are not parallel to each other. In this case, a warping correction can be applied as shown in Figure S2. The same warping parameters generated from the standard sample (such as a Ag foil) can be applied to other Ag containing materials using the same crystal.

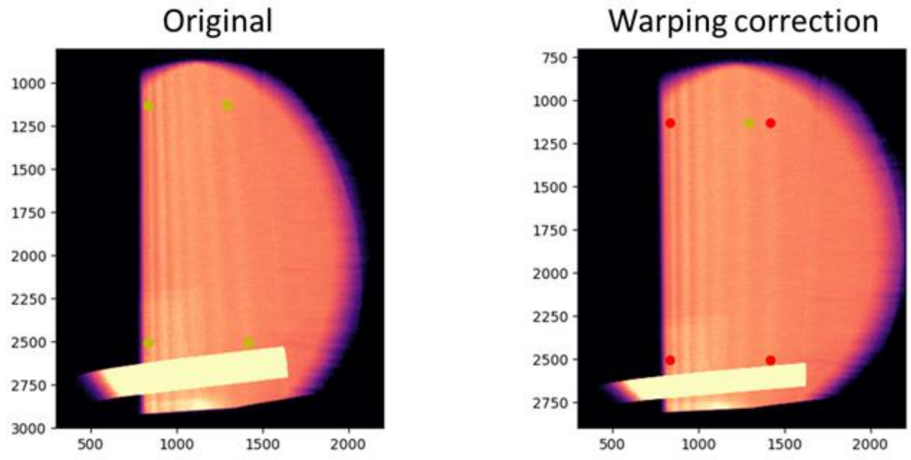

Figure S2 Four points are chosen manually (see yellow points) on the fringes of the original absorption image. Then the fringes are automatically aligned by transforming yellow points positions to red points positions.

S3 Results with different total exposure times

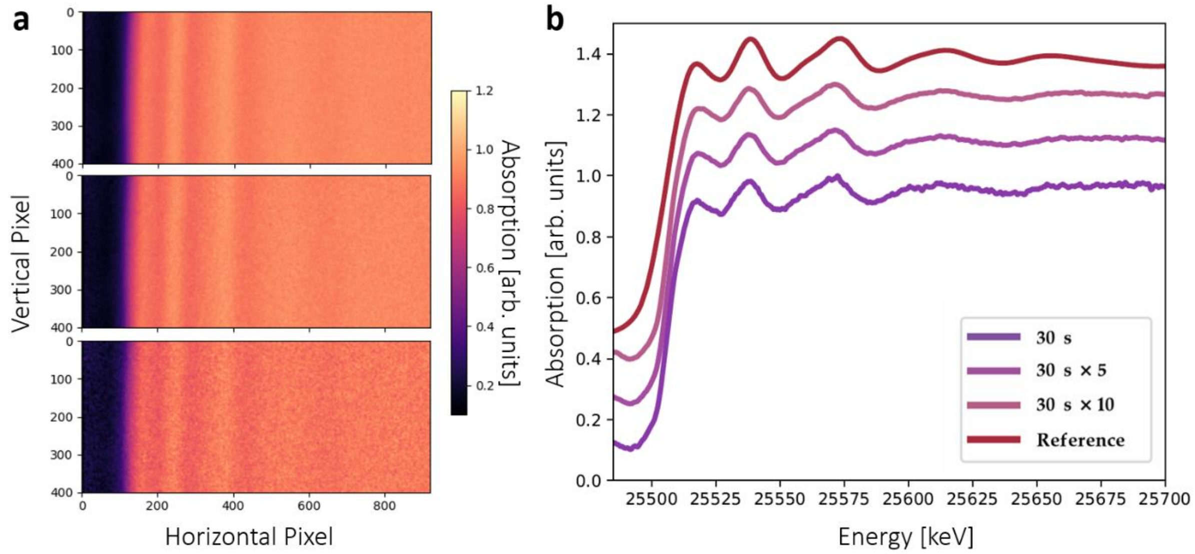

Figure S3 (a) Ag foil absorption images of averaging 10 frames, 5 frames and 1 frame (from top to bottom) (b) XANES spectra for averaging different number of frames

The XANES results that have been shown in the main manuscript were averages of 10 frames of 30 s exposure time for I_s and I_0. Figure S2 shows the absorption images and the corresponding spectra of Ag using the same Si(311) reflection with averaging different number of frames. Also for a total exposure time down to 1 minute (30 s with the sample and 30 s without sample), the spectrum is still in good agreement with a reference spectrum taken at the synchrotron.

S4 EXAFS measurement

The EXAFS spectrum in the main manuscript was obtained by stitching two absorption images measured for two angles with a Si(311) diffraction order. These two images are shown in Figure S4.1 (a) and (b).

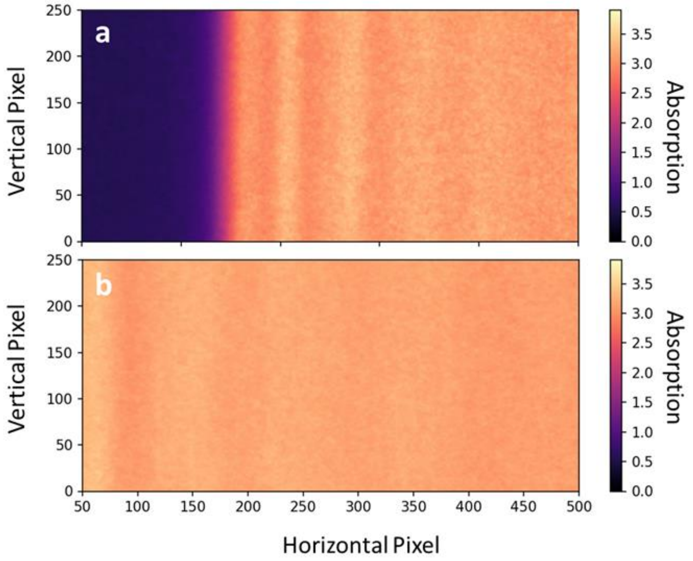

Figure S4.1 (a) Absorption image measured at the crystal angle covering the K-edge. (b) Another absorption image measured after rotating the crystal by 0.05 degree.

The limitation of the current method is that distinct features are needed in the second image and some spectrum overlap (e.g., at least 1 peak/feature in common) is required in order to stitch them together. However, there are also other measurement methods instead of stitching two spectra, which can overcome this limitation:

1. To use other diffraction orders such as Si(111) and adjust the bending curvature, which increases the maximum energy range in a single image, but at the cost of a degraded energy resolution. However, since the primary signal of EXAFS mainly arises from the low frequency components, the energy resolution does not have to be as high in EXAFS as it needs to be in XANES. Switching between Si(311) and Si(111) diffraction orders is feasible by simply rotating the crystal. Therefore, one can combine both

high resolution and lower resolution, e.g., to measure XANES by using the Si(311) diffraction order and then rotate the crystal to the Si(111) diffraction order for the EXAFS measurement.

2. Instead of rotating to just one other angle and thus measuring two spectra, which are shifted with respect to each other by a large fraction of the maximum energy range and therefore might be difficult to stitch, a scanning fashion can be adopted (see example in Figure S4.2). The Laue crystal (bending radius ~11 m, Si(311) diffraction order) was rotated in very fine steps of 0.001 degree per step, 100 steps in total. At each step, the energy range is extended by a small fraction (~ 3 eV, 1.2 % of maximum energy range) with a big fraction of the energy range overlapping with the previous one. The scanning with fine steps not only extends the energy range smoothly, but also increases the data statistics in the overlapping regions. Therefore, there is no need to average multiple image frames at each step to increase the statistics (the spectra shown in the main manuscript are averaged of 10 image frames). If better optimized fine steps are chosen e.g., 10 steps in total, the total acquisition time is unchanged.

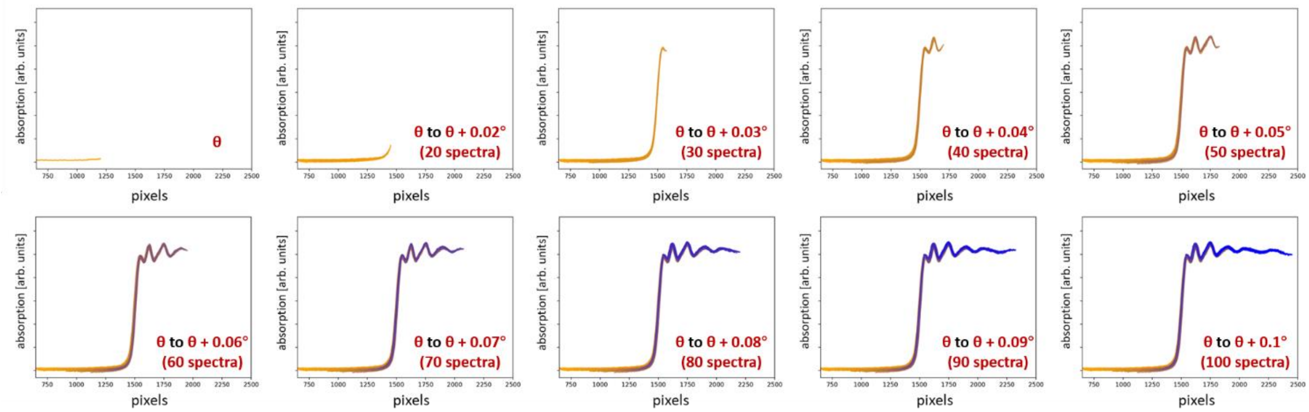

Figure S4.2 Extending the energy range by rotating the crystal in fine steps. In this example, we used the same Si(311) diffraction order of the Laue crystal (bending radius ~11 m, maximum energy range ~250 eV). The crystal was rotated in total by 0.1 degree in 100 steps (30 s exposure time at each step for both the I_s and I_0). Each spectrum at each step was shifted by a linear function. The final overlapped 100 spectra (the last figure) were not calibrated or normalized, illustrating the stability and feasibility of the scanning fashion.

Finally, regarding the EXAFS energy range, it should be mentioned that the x-ray intensity of the 26 keV energy configuration drops quickly after 26 keV and there is no flux left above 26.5 keV (see Figure S4.3). For Ag whose K-edge is at ~25.5 keV, the energy range for EXAFS can be further extended by switching to another MuCLS energy configuration e.g., 27 keV. Some preliminary tests have been done on fast

energy configuration switching lately, during which 1 keV energy changes could be realized in less than 5 s.

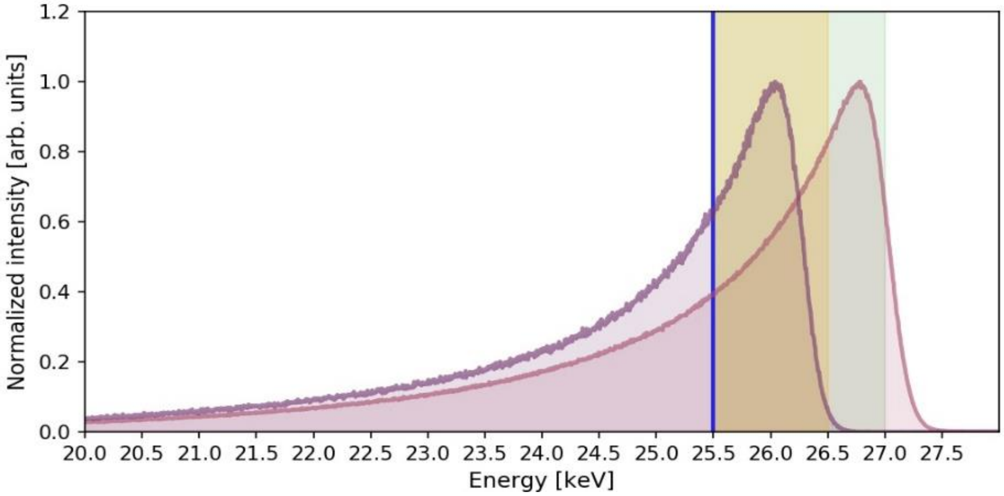

Figure S4.3 MuCLS measured source spectrum of 26 keV and 27 keV configurations (measured with energy dispersive detector, KETEK AXAS-D, KETEK GmbH, Munich, Germany). The blue line highlights the Ag K-edge. Yellow and green regions correspond to 1 keV, 1.5 keV energy range, respectively.

Reference

[1] http://skuld.bmsc.washington.edu/scatter/AS_periodic.html

[2] https://iupac.org/what-we-do/periodic-table-of-elements/
